# Spatial Distribution and Factors Associated with Khat Chewing among Adult Males 15-59 Years in Ethiopia Using a Secondary Analysis of Ethiopian Demographic and Health Survey 2016: Spatial and Multilevel Analysis

**DOI:** 10.1155/2020/8369693

**Published:** 2020-04-20

**Authors:** Zemenu Tadesse Tessema, Tadele Amare Zeleke

**Affiliations:** ^1^Department of Epidemiology and Biostatics, Institute of Public Health College of Medicine and Health Sciences, University of Gondar, Gondar, Ethiopia; ^2^Department of Psychiatry, School of Medicine, College of Medicine and Health Science, University of Gondar, Gondar, Ethiopia

## Abstract

**Background:**

Khat chewing has become prevalent in the world due to the improvement of road and air transportation. In Ethiopia, khat chewing is more prevalent and widely practiced by men. Khat has a negative effect on social, economic, and mental health. There is variation in khat cultivation, use, and factors that associated with khat chewing in the Ethiopian regions. Therefore, this study is aimed at showing spatial distribution and factors associated with khat chewing among male adults 15-59 years in Ethiopia.

**Methods:**

A total of 12,594 men were included in this study. ArcGIS version 10.7 software was used to show the spatial distribution of chewing khat among adult men in Ethiopia. The Bernoulli model was applied using Kilduff SaTScan version 9.6 software to identify significant purely spatial clusters for chewing khat in Ethiopia. A multilevel logistic regression model was fitted to identify factors associated with khat chewing. A *P* value < 0.05 was taken to declare statistically significant predictors.

**Results:**

The EDHS 2016 survey showed that the high proportion of chewing khat was found in Dire Dawa, Harari, Southern Oromia, Somali, and Benishangul Gumuz regions. In spatial scan statistics analysis, a total of 126 clusters (LLR = 946.60, *P* value < 0.001) were identified. Age group 30-44 years old (AOR = 1.60, 95% CI: 1.37, 1.86) and 45-59 years old (AOR = 1.33, 95% CI: 1.09, 1.61), being single (AOR = 1.86, 95% CI: 1.64, 2.12), Muslim religion followers (AOR = 15.03, 95% CI: 11.90, 18.90), media exposed (AOR = 0.77, 95% CI: 0.68, 0.86), had work (AOR = 2.48, 95% CI: 2.08, 2.95), alcohol drinker (AOR = 3.75, 95% CI: 3.10, 4.53), and region (Afar, Amhara, Benishangul Gumuz, Gambela, Harari, Oromia, Somali, Southern Nations, Nationalities, and People's Region (SNNPR), and Tigray) and two cities (Addis Ababa and Dire Dawa) were statistically significant factors affecting chewing khat in Ethiopia.

**Conclusions:**

In Ethiopia, the spatial distribution of khat chewing among adult men was nonrandom. A high proportion of khat chewing was observed in Dire Dawa, Harari, Southern Oromia, Somali, and Benishangul Gumuz regions. Older age group, being single marital status, alcohol drinker, media unexposed, had no work, and Muslim religion follower were factors affecting khat chewing. Policymakers should be given spatial attention in reducing the prevalence of chewing khat by teaching the health impact of khat chewing through media in the identified regions.

## 1. Background

Khat is an evergreen shrub cultivated as a bush or small tree native to Ethiopia, East Africa, and the southern Arabian Peninsula [1]. Its young buds and tender leaves contain amphetamine-like psychoactive substances, which produce euphoria and stimulation [2]. In Yemen and the Jazan region of Saudi Arabia, the prevalence of khat chewing has been high. According to global synthetic drug assessment in 2014, in Yemen, the prevalence of khat chewing among people aged 16 and above was 52% [3].

Globally, even though the exact number is not known, about 5 to 10 million people use khat in the world [4]. According to the United Nations, Office on Drugs and Crime reported that about 72% of Yemeni men and 32.6% of women used khat in 2006 [5]. In other study, chewing khat among Yemeni men and women was 90% and 60%, respectively [6]. In Somalis living in London, the prevalence of khat chewing in a week was 67% [7]. In Saudi Arabia, the prevalence of current khat use among men was 36.9% [8].

Based on EDHS 2016, the prevalence of ever chewed khat was 12% and 27% of women and men, respectively [9]. Khat consumption increases with age and peaks at age 30-34 among both women (15%) and men (34%) [9].

Khat chewing had an impact on physical health (loss of teeth, gum disease, and mouth problems); constipation; stomach problems; weight loss; sleeplessness; cardiovascular problems such as heart attacks, high blood pressure, and stroke; diabetes; respiratory problems; male impotence; and bowel cancer from the chemicals sprayed on khat [5, 10–12]. Reasons for khat use were peer pressure, academic performance, alertness, feeling excitement, and well-being [13, 14].

The major predisposing factors for khat chewing include a family member who had a history of chewing khat [15, 16], the reason for concentration and relaxation [15, 17], alcohol drunker [18], Muslim religion follower, age 45 to 49, and attended mass media [19–21]. In Ethiopian regions, Oromia, Southern Nations, Nationalities, and People's Region (SNNPR), Gambela, Harari, and Dire Dawa were also significant factors for khat use [20].

There is variation in khat cultivation, use, and factors that associated with khat chewing in the Ethiopian regions. Even though different studies tried to show the predisposing factors for khat chewing in Ethiopia, the spatial distribution of khat chewing was not done before. Identifying the spatial distribution and factors of khat chewing in Ethiopia can help health planners and policymakers for specific interventions to decrease khat chewing.

Therefore, this study is aimed at assessing the spatial distribution and factors associated with khat chewing among adults aging 15-59 years in Ethiopia.

## 2. Methods

### 2.1. Study Design, Period, and Setting

**Figure 1:**
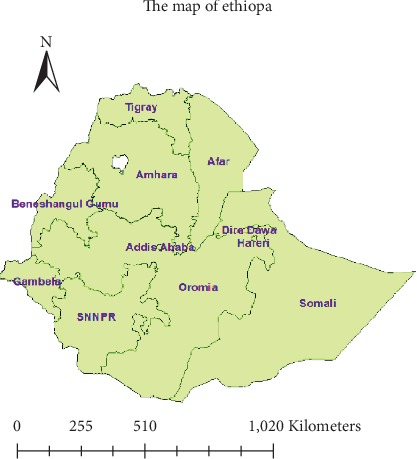
Study areas of nine regions and two city administrations in Ethiopia, EDHS 2016.

A population-based cross-sectional study was conducted from January 18 to June 27, 2016. Ethiopia is located in the horn of Africa. It has a total area of 1,100,000 km^2^ and lies between latitudes 3° and 15°N and longitudes 33° and 48°E. Ethiopia has nine regions (Afar, Amhara, Benishangul Gumuz, Gambela, Harari, Oromia, Somali, Southern Nations, Nationalities, and People's Region (SNNPR), and Tigray) and two administrative cities (Addis Ababa and Dire Dawa) (Figure 1).

### 2.2. Source and Study Population

The source population was all men from 15 to 59 years in Ethiopia. The study population was all men aging from 15 to 59 in the selected enumeration areas. All men aging 15-59 who had interviewed about ever chewed khat were included in the study. However, respondents with missing data for the outcome variable were excluded from the study.

### 2.3. Sample Size and Sampling Procedure

A total of 12,594 men were included in this study. Weighted values were used to restore the representatives of the sample data. Sample weights were calculated in each Men's Record (MR) EDHS datasets. The survey covered all nine regions and the two city administrations of Ethiopia. Participants were selected based on a stratified two-stage cluster sampling technique in each survey year EDHS 2016. After excluding clusters with no recorded x and y coordinates, a total of 621 clusters in 2016 were encompassed in this study. The detail sampling procedure was available in each EDHS reports from measure DHS website (http://www.dhsprogram.com).

### 2.4. Data Collection Tools and Procedures

The data were obtained from the Ethiopian Demographic and Health Surveys (EDHS) Program by requesting for this work and accessing ht6 were encompassed http://www.dhsprogram.com website. Ethiopian Demographic and Health Survey data were collected by two-stage stratified sampling. Each region of the country was stratified into urban and rural areas.

## 3. Variables

### 3.1. Outcome Variables

The outcome variable is taken as binary response men who ever chewed khat in his life coded as “1” and men who do not chew chat in his life is coded as “0.”

### 3.2. Independent Variables

From the WR EDHS dataset, all sociodemographic, socioeconomic, and lifestyle variables (individual and community level) were taken as independent in this study. Only two variables residence and region were collected at the community level. The individual-level variables considered the following: age group, religion, marital status, educational status, alcohol drinking, and media exposure (if men use at least one from the three taken as media exposed: reading a newspaper, reading magazines, and watching television); wealth ranking was grossly categorized into 5 major quintiles lowest (poorest), second (poorer), middle (middle), fourth (richer), and highest (richest), which we recategorized into three “poor (poorest+poor), middle, and rich (richest+rich)” for ease of analysis. The independent variables were screened based on different literatures [16, 17, 19, 20, 22–24].

### 3.3. Operational Definition

Ever khat chewer is defined as a respondent whoever chewed khat during his lifetime.

Alcohol drinking is defined as a respondent who drank alcohol during one month preceding the survey at least once per month.

#### 3.3.1. Data Management

STATA version 14.1, excel, and ArcGIS 10.1 were used for statistical and spatial analysis, respectively. Sample weighting was done before further analysis.

## 4. Analysis

### 4.1. Geospatial Analysis

#### 4.1.1. Spatial Distribution of Khat Chewing in Ethiopia

Among a total of 643 clusters, 621 were considered for the spatial analysis of khat chewing in Ethiopia (21 clusters dropped because of no coordinate data in the cluster). Each point on the map represents one enumeration area with a prevalence of khat chewing in each cluster.

#### 4.1.2. Spatial Autocorrelation Analysis

The spatial autocorrelation (Global Moran's I) statistic measures whether the khat chewing patterns were dispersed, clustered, or randomly distributed in the study area [25]. “Moran's I” is a spatial statistics used to measure spatial autocorrelation by taking the entire dataset and produce a single output value which ranges from -1 to +1. Moran's I values close to −1, 1, and 0 indicate disease dispersed, disease clustered, and disease distributed randomly, respectively. A statistically significant Moran's I (*P* < 0.05) leads to rejection of the null hypothesis (khat chewing is randomly distributed) and indicates the presence of spatial autocorrelation.

#### 4.1.3. Incremental Autocorrelation

Measuring spatial autocorrelation for a series of distances optionally creates a line graph of those distances and their corresponding*Z*-scores.*Z*-scores show the intensity of spatial clustering and statistical significance. Peak *Z*-scores show distances where spatial processes promoting clustering are most pronounced. These peak distances are often proper values to use for tools with a Distance Band or Distance Radius parameter. This tool can help to select an appropriate distance threshold or radius for tools that have these parameters, such as hot spot analysis [25].

#### 4.1.4. Hot Spot Analysis (Getis-OrdGi^∗^ Statistic)

Getis-OrdGi^∗^ statistic was computed to measure how spatial autocorrelation varies over the study site by calculating GI^∗^ statistic for each area. *Z*-score is computed to find the statistical significance of clustering, and the *P* value is computed for the significance. Statistical output with high GI^∗^ indicates “hotspot” whereas low GI^∗^ means a “cold spot.”

#### 4.1.5. Spatial Scan Statistical Analysis

A Bernoulli-based model was used in which events at particular places were analyzed if adults whether chewing chat or not coded as 1/0 variable. The scan statistics developed by Kulldorff and SaTScan™ software version 9.6 were used to identify the presence of purely spatial home deliver clusters. Scan statistics did scanning gradually across the space to identify the number of observed and expected observations inside the window at each location. The scanning window with the maximum likelihood was the most likely high performing clusters, and a *P* value was assigned to this cluster.

#### 4.1.6. Spatial Interpolation

The spatial interpolation technique is used to predict khat chewing for unsampled areas based on sampled EAs. For the prediction of unsampled EAs, we used the deterministic and geostatistical ordinary Kriging spatial interpolation technique using ArcGIS 10.7 software.

### 4.2. Statistical Analysis

#### 4.2.1. Model Building

We fit four models: the null model without predictors, the model I with only individual-level variables, model II with only community-level variables, and model III both individual-level and community-level variables. These models were fitted using a STATA command *melogit*. For model comparison, we used the log-likelihood ratio (LLR) and deviance. The highest log-likelihood or the smallest deviance wins the best-fitted model. Therefore, model III which includes both individual and community-level variables was selected as the best fit model for the data.

#### 4.2.2. Parameter Estimation Method

The fixed effects (a measure of association) were used to estimate the association between the likelihood of khat chewing and explanatory variables at both community and individual levels and were expressed as odds ratios with 95% confidence interval. Regarding the measures of variation (random-effects), the intracluster correlation coefficient (ICC), proportional change in community variance (PCV), and median odds ratio (MOR) were used.

The aim of the median odds ratio (MOR) is to translate the area level variance in the widely used odds ratio (OR) scale, which has a consistent and intuitive interpretation. The MOR is defined as the median value of the odds ratio between the area at the highest risk and the area at the lowest risk when randomly picking out two areas. The MOR can be conceptualized as the increased risk that (in median) would have if moving to another area with a higher risk.

It is computed by MOR = exp[√(2 × VA) × 0.6745] [26], where VA is the area level variance and 0.6745 is the 75th percentile of the cumulative distribution function of the normal distribution with mean 0 and variance 1. See elsewhere for more detailed explanation [26]. The proportional change in variance is calculated as PCV = [(VA − VB)/VA]∗100 [27], where VA is the variance of the initial model and VB is the variance of the model with more terms.

## 5. Results

A total of 12,594 participants were comprised in the analysis. The prevalence of ever khat chewing in this study was 3418 (27.14%) with 95% CI 26.37% to 27.92%. Almost half of participants, 6426 (51.03%), were in the age of 15-29 years. The majority of the participants 10,098 (80.18%) were from rural, and most of them 5876 (46.66%) were in the primary education class. The median age of the respondent was 29 with an interquartile range (IQR) of 21-39. Around two-thirds of 7705 (61.17%), of participants, were married (Table 1).

### 5.1. Spatial Analysis Result

#### 5.1.1. Spatial Distribution of Khat Chewing

**Figure 2:**
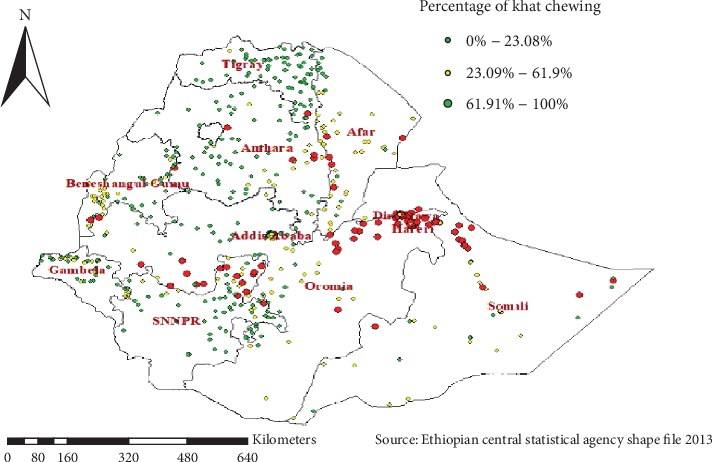
Spatial distribution of khat chewing among adults aging 15-59 across regions of the country, EDHS 2016.

As shown in Figure 2, a high proportion of chewing khat was observed in Dire Dawa, Harari, Southern Oromia, Somali, and Benishangul Gumuz regions which ranges from 61.9% to 100%. The low proportion of khat chewing was observed in Amhara and some parts of Oromia and Tigray regions of Ethiopia.

#### 5.1.2. Spatial Autocorrelation of Khat Chewing

**Figure 3:**
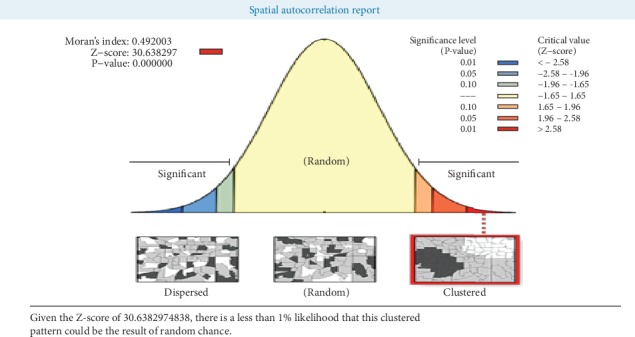
Spatial autocorrelation of khat chewing among adults aging 15-59 across regions of the country, EDHS 2016.

In the EDHS 2016 survey, the spatial distribution of khat chewing in Ethiopia was nonrandom. The global Moran's I value was 0.49 (*P* value < 0.001) with a *Z*-score value of 30.63 with that there is a less than 1% likelihood that this clustered pattern could be the result of chance (Figure 3).

#### 5.1.3. Incremental Spatial Autocorrelation Khat Chewing

**Figure 4:**
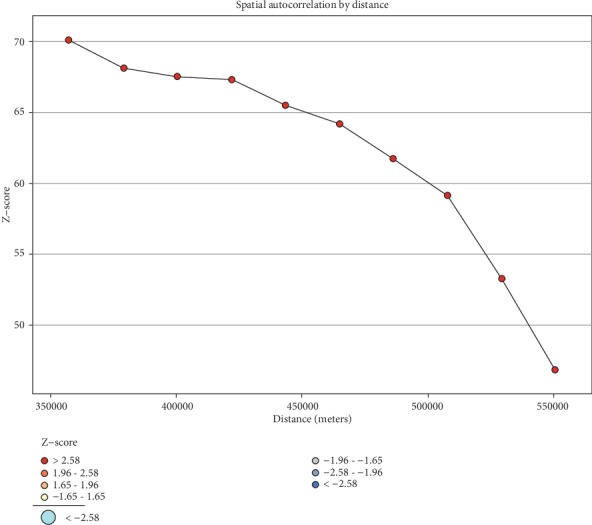
Spatial incremental autocorrelation of chewing khat in Ethiopia, EDHS 2016.

To determine spatial clustering for khat chewing, global spatial statistics were estimated using Moran's I value. As shown in Figure 4 below, a statistically significant *Z*-score indicated at 357.42 km distances, where spatial processes promoting clustering are most pronounced. The incremental spatial autocorrelation indicated that a total of 10 distance bands were detected with a beginning distance of 357,428.11 meters.

#### 5.1.4. Hot Spot Analysis (Getis-OrdGi^∗^ Statistic)

**Figure 5:**
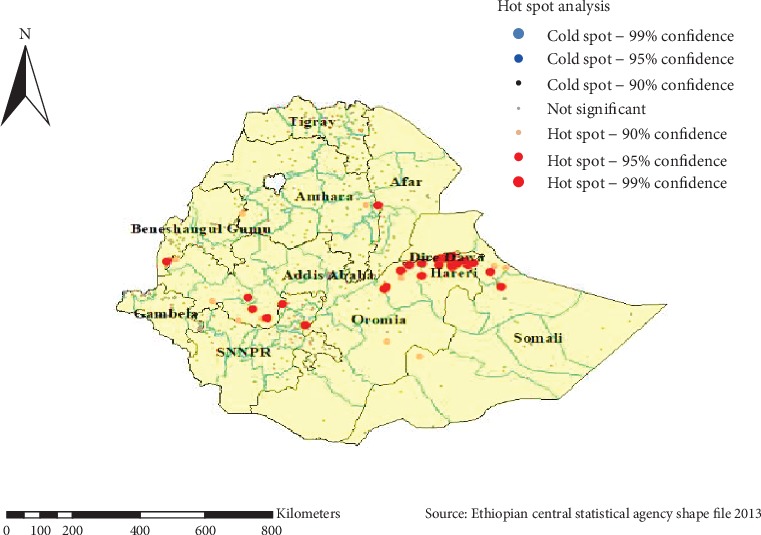
Hot spot analysis of khat chewing in Ethiopia, EDHS 2016.

As shown in Figure 5 below, the red color indicated the more intense clustering of high (hot spot) proportion of khat chewing in the five years preceding the survey period and observed in Dire Dawa, Harari, Southern Oromia, Somali, and Benishangul Gumuz regions.

#### 5.1.5. Spatial Scan Statistical Analysis

**Figure 6:**
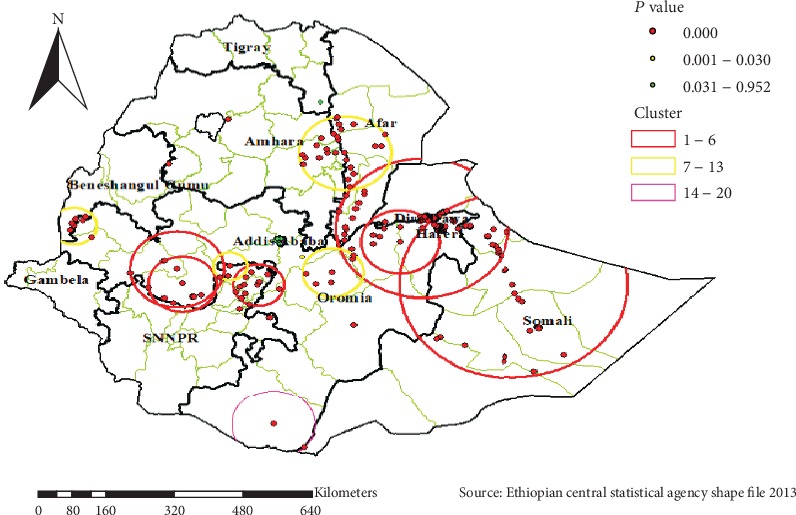
Spatial scan statistics of khat chewing in Ethiopia, EDHS 2016.

As shown in Figure 6 and Table 2 below, the red window indicated the identified significant clusters inside the window. In spatial scan statistics, a total of 140 most likely clusters were identified in EDHS 2016 survey. The most likely clusters of khat chewing were detected in most parts of Harari, Dire Dawa, southwestern part of Oromia, SNNPR, and Somali regions. Among the most likely clusters, 126 of them were primary clusters which are located at 9.303717 N, 41.792390 E with a 200.29 km radius (log‐likelihood ratio (LLR) = 480.60, *P* value < 0.001).

#### 5.1.6. Spatial Interpolation

**Figure 7:**
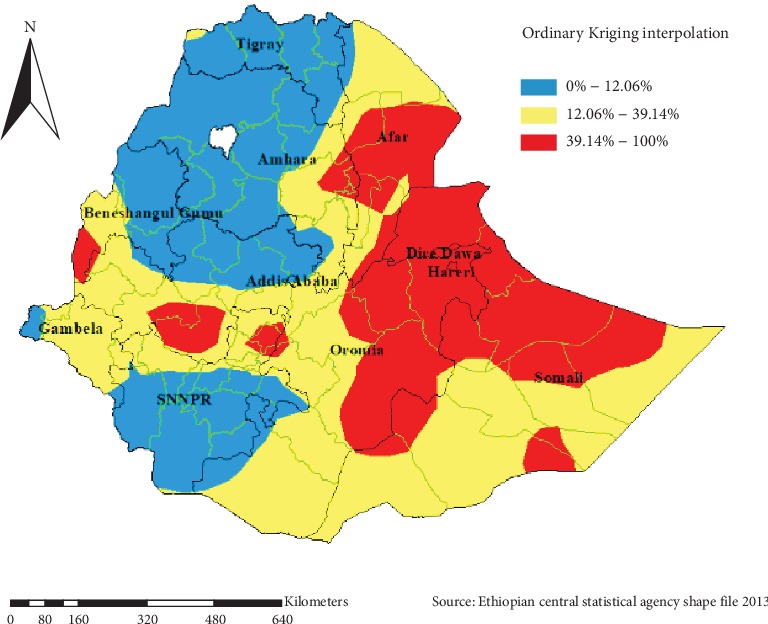
Spatial interpolation of khat chewing among males in Ethiopia, EDHS 2016.

We used ordinary Kriging geostatistical interpolation for the prediction of khat chewing prevalence of unsampled areas. Based on geostatistical Kriging analysis, in 2016 EDHS, exclusively Dire Dawa, Harari, some parts of Oromia, Somali, and some parts of SNNPR had a prevalence of less 39.14% to 100% (Figure 7).

### 5.2. Multilevel Logistic Regression Analysis

#### 5.2.1. The Random-Effects Analysis Result

The fixed effects (a measure of association) and the random intercepts for khat chewing are presented in Table 2. The results of the empty model (model I) revealed that there was statistically significant variability in the odds of khat chewing with community variance (*τ* = 4.73, *P* value ≤ 0.001). Similarly, the ICC in the empty model implied that 59.02% of the total variance in the khat chewing was attributed to differences between communities (residence and region). The community variance was expressed as the intracluster correlation coefficient (ICC) and the median odds ratio (MOR). Moreover, the MOR was 4.6 (95% CI 3.20, 6.58) which implied that the odds of having chewing khat was 4.6 times higher when men moved from low- to high-risk communities. This showed the existence of significant heterogeneity in khat chewing across different communities. In the full model (model adjusted for both individual- and community-level factors), community variance (community variance = 1.60; SE 0.15; *P* value ≤ 0.001) remained significant but reduced. About 36.84% of the total variance of chewing khat that can be attributed to the contextual-level factors remained significant even after considering some contextual risk factors. The proportional change in variance (PCV) in this model was 66.17% which showed that 66.17% of community variance observed in the null model was explained by both community- and individual-level variables (Table 3).

#### 5.2.2. Fixed-Effects Analysis Result

In the bivariate analysis, age group, household headed, marital status, residence, religion, educational status, region, wealth index, media exposure, working status, and alcohol drinking were associated factors with khat chewing at a *P* value of less than or equal to 0.2. Consequently, these variables were subjected to multivariable analysis, and it was noted that age group, marital status, religion, region, media exposure, working status, and alcohol drinking were statistically significant variables associated with khat chewing at a *P* value of 0.05.

The odds of chewing khat among Ethiopian male adults age group 30-44 and 45-59 increase by 60% and 33% as compared to age group 15-29 years (AOR = 1.60, 95% CI: 1.37, 1.86; AOR = 1.33, 95% CI: 1.09, 1.61, respectively). Being single increases the odds of chewing khat by 86% as compared to married adults (AOR = 1.86, 95% CI: 1.64, 2.12). The odds of chewing khat among Muslim religion followers were 15.06 times higher as compared to Orthodox religion followers (AOR = 15.09, 95% CI: 11.94, 18.97). The odds of chewing khat increase by 2.48 times higher among men who had work as compared to their counterparts (AOR = 2.48, 95% CI: 2.08, 2.95). The odds of chewing chat decrease by 33% among media exposed male adults as compared to nonexposed (AOR = 0.77, 95% CI: 0.68, 0.86). The likelihood of chewing chat among alcohol drinkers was 3.75 times higher as compared to nondrinkers (AOR = 3.75, 95% CI: 3.110, 4.53). The odds of khat chewing vary in region (Table 3).

## 6. Discussion

This study showed the spatial distribution and factors associated with khat chewing among male adults in Ethiopia by using the Ethiopian Demographic and Health Survey 2016. The study revealed that khat chewing was more common in Dire Dawa, Harari, Southern Oromia, Somali, and Benishangul Gumuz regional states of Ethiopia. Age group, marital status, religion, working status, media exposure, and alcohol drinking were identified as associated factors with khat chewing in Ethiopia.

This study revealed that the spatial distribution of khat chewing in Ethiopia was nonrandom. Khat chewing was highly clustered in Dire Dawa, Harari, Southern Oromia, Somali, and Benishangul Gumuz regional state of Ethiopia. In line with this, high proportion clustering, spatial scan statistic analysis revealed that 126 significant clusters were found.

In this finding, different factors of khat chewing were assessed by the logistic regression model. Individuals who are residents of Afar, Amhara, Oromia, Somali, Benishangul Gumuz, SNNPR, Gambela, Harari, Addis Ababa, and Dire Dawa had higher odds of khat use when compared with Tigray. This study is in line with spatial analysis of the result of Figure 4; the reason might be due to the fact that nowadays khat is transported throughout Ethiopia due to the expansions of both land and air transportation. The powerful force has perhaps increased market prospects and prices of khat [20].

When compared with people less than 15-29 years of age, all older (30-44 and 45-59) people have a higher risk of being a khat chewer. This finding is in line with other studies [23, 24]. The possible reason could be young people tend to be under family control which reduces their risk of being a chat chewer.

In this study, Muslim religion followers were at a higher risk of being khat chewer when compared to Orthodox Christians. This finding is supported by the research done in Chiro town [20], Dera woreda [21], and Butajira [17]. This could be due to the fact that khat use was acceptable in the sociocultural and functional purposes with a restriction of the frequency, amount, and type of khat [18]. Another reason might be to get the maximum concentration level during praying, to increase socialization, for confirming norms, and for stabilizing emotions [17].

In this study, those men who are single increased odds of khat chewing 2.48 times than married. This might be due to the reason that individuals who are single are high risk for substance use and unmarried individuals are positively associated with khat chewing [28].

The odds of khat chewing were increased by 2.48 times higher among individuals who have work when compared with counterparts. Perhaps, the working individuals may enable to use khat than individuals who had no work who spent their time with khat chewing.

In our study, the odds of khat chewing were decreased by 33% among those who attend mass media as compared to those who did not attend. This could be due to the fact that individuals who read magazines and newspapers, listen to the radio, and watch television had the awareness of the bad result of khat use, like depression, anxiety, sleep disorder, conflict in the family, reduced appetite, and neurological and dental problems, making them less likely to chew khat [29–32].

The odds of khat chewing are increased by 3.75 times higher among alcohol drinkers when compared with a nondrinker. This is consistent with another finding; harmful drinking is common among khat users that account for 53.9% [33]. The possible reason might be that alcohol drinking is used to break the stimulating effect of khat; therefore, alcohol is helpful for sleep and used as self-treatment from khat-induced distress [18].

We have also found a significant difference in the risk of khat chewing among the regions of Ethiopia with the Tigray region the least likely region to chew khat. This finding is in line with another study conducted in Gondar, Ethiopia [34]. This could be due to the cultural difference and political instability in the high-risk areas of khat chewing like Ethiopian Somali which leads to stress.

This study has strengths of nationally representative data, and advanced statistical models were used to account correlations within clusters. However, this study has limitations of the cross-sectional nature of the study, which may not indicate true causality. Besides, the effects of the health system and health worker factors were not assessed during data collection.

## 7. Conclusions

In Ethiopia, the spatial distribution of khat chewing among adult men was nonrandom. A high proportion of khat chewing was observed in Dire Dawa, Harari, Southern Oromia, Somali, and Benishangul Gumuz regions. Older age group, being single, alcohol drinker, media unexposed, had no work, and Muslim religion followers were factors affecting khat chewing. Policymakers should be given spatial attention in reducing the prevalence of chewing khat by teaching the health impact of khat chewing through media in the identified regions.

## Figures and Tables

**Table 1:** Individual and community characteristics of adult men 15-59 years in Ethiopia, EDHS 2016.

Variable	Frequency (*n* = 12,594)	Percentage
Ever chewed khat		
Yes	3418	27.14
No	9176	72.86
Age group		
15-29	6426	51.03
30-44	4173	33.13
45-59	1995	15.84
Sex of the household		
Male	11034	87.61
Female	1560	12.59
Marital status		
Married	7705	61.17
Single	489	38.83
Residence		
Urban	2496	19.82
Rural	10098	80.18
Religion		
Orthodox	5677	45.07
Muslim	3916	31.09
Protestant	2745	21.80
Others^∗^	256	2.03
Education		
No education	3773	29.06
Primary	5876	46.66
Secondary	1846	14.66
Higher	1099	8.73
Region		
Tigray	795	6.31
Afar	82	0.65
Amhara	3206	25.46
Oromia	4713	37.43
Somali	326	2.59
Benishangul Gumuz	123	0.98
SNNPR	2886	20.53
Gambela	36	0.29
Harari	31	0.25
Addis Ababa	196209	4.93
Dire Dawa	71	0.57
Media exposure		
Yes	8154	64.74
No	4440	35.26
Wealth index		
Poor	4272	33.92
Middle	2427	19.27
Rich	5895	46.81
Working status		
Yes	11172	88.71
No	1422	11.29
Alcohol drinking		
Yes	5873	46.64
No	6721	53.36

Note: others^∗^ = Catholic, cultural belief.

**Table 2:** SaTScan analysis of khat chewing among men in the last five years in Ethiopia, EDHS 2016.

Cluster type	Significant enumeration areas (clusters) detected	Coordinates/radius	Populations	Cases	RR	LLR	*P* value
Primary	453, 557, 441, 594, 166, 30, 473, 380, 74, 311, 613, 273, 151, 631, 535, 519, 282, 471, 202, 111, 607, 43, 5, 644, 173, 25, 443, 185,352, 606, 444, 467, 115, 390, 546, 614, 190, 363, 27, 393, 383, 385, 514, 610, 179, 224, 28, 493, 60, 228, 101, 133, 29, 56, 397, 140, 500, 238, 157, 257, 418, 58, 329, 580, 240, 396, 513, 387, 534, 44,523, 587, 242, 495, 483, 194, 321, 281, 381, 288, 357, 68, 419, 454, 501, 212, 436, 642, 93, 622, 372, 1, 307, 566, 186, 491, 333, 8, 210,412, 64, 57, 439, 506, 476, 277, 568, 527, 564, 39, 336, 135, 22, 37, 122, 51, 245, 116, 230, 49, 71, 239, 529, 251, 573, 33, 484, 102, 214	(9.303717 N, 41.792390 E)/200.29 km	1110	955	4.02	946.56	≤0.001
Secondary	372, 93, 412, 333, 476, 506, 453, 491, 441, 557, 594, 30, 25, 166	(8.949349 N, 41.312400 E)/91.51 km	496	453	3.73	480.60	≤0.001

**Table 3:** Multivariable multilevel logistic regression analysis of both individual- and community-level factors associated with khat chewing in Ethiopia, EDHS 2016.

Individual- and community-level variables	Models			
	Null model	Model I	Model II	Model III
	AOR (95% CI)	AOR (95% CI)	AOR (95% CI)	AOR (95% CI)
Men age				
15-20 years		1		1
20-34 years		1.67 (1.43, 1.94)		1.60 (1.37, 1.86)^∗^
35-49 years		1.38 (1.13, 1.67)		1.33 (1.09, 1.61)^∗^
Household head				
Male		1		1
Female		1.03 (0.88, 1.20)		0.99 (0.85, 1.16)
Marital status				
Had a partner		1		1
Not having a partner		2.08 (1.55, 2.33)		1.86 (1.64, 2.12)^∗^
Religion				
Orthodox		1		1
Muslim		16.1 (12.74, 20.35)		15.06 (11.9, 18.9)^∗^
Protestant		0.48 (0.36, 0.64)		0.43 (0.34, 1.05)
Others		0.89 (0.52, 1.52)		0.77 (0.46, 1.29)
Men education				
Unable to read and write		1		1
Primary education		1.16 (0.99, 1.37)		1.10 (0.93, 1.29)
Secondary education		1.26 (1.02, 1.55)		1.09 (0.89, 1.35)
Higher education		1.37 (1.10, 1.74)		1.17 (0.94, 1.47)
Men occupation				
Not working		1		1
Working		2.24 (2.08, 2.94)		2.48 (2.08, 2.95)^∗^
Media exposure				
No exposed		1		1
Exposed		0.86 (0.68, 0.95)		0.77 (0.65, 0.87)^∗^
Wealth index				
Poor		1		1
Middle		0.99 (0.80, 1.22)		0.98 (0.79, 1.22)
Richer		1.24 (1.02, 1.50)		1.00 (0.82, 1.23)^∗^
Alcohol drinking				
No		1		
Yes		3.45 (2.84, 4.18)		3.75 (3.10, 4.53)^∗^
Residence				
Urban			1	1
Rural			1.06 (0.74, 1.53)	0.78 (0.55, 1.10)
Region				
Tigray				1
Afar			24.87 (12.13, 50.99)	8.36 (4.52, 16.49)^∗^
Amhara			2.85 (1.42, 5.70)	2.04 (1.11, 3.74)^∗^
Oromia			30.11 (15.45, 58.66)	20.10 (11.09, 36.44)^∗∗∗^
Somali			38.30 (19.40, 75.62)	12.34 (6.63, 22.95)^∗^
Benishangul Gumuz			8.58 (4.15, 17.75)	4.03 (2.12, 7.67)^∗^
SNNPR			5.12 (2.59, 10.15)	11.48 (6.1, 21.2)^∗^
Gambela			10.59 (5.13, 21.88)	19.48 (10.1, 37.2)^∗^
Harari			208 (97.5, 445)	122 (61, 241)^∗^
Addis Ababa			23.31 (11.15, 48.71)	15.48 (8.37, 29.97)^∗^
Dire Dawa			110 (5.93, 233.17)	58.31 (30.0, 113.1)^∗^
*Random effects*				
ICC%	59.02	49.20	43.36	32.84
PCV%	1	32.76	46.93	66.17
MOR	7.96	5.47	10.96	4.60
Model fitness				
Log-likelihood ratio	-5729	-4955	-5567	-4783
Deviance	11458	9910	11134	9566

NB: ∗ = significant at *P* value = 0.05; CI = confidence interval; AOR = adjusted odds ratio; others = traditional religion followers.

## Data Availability

The data used to support the findings of this study are available from the corresponding author upon request.

## Abbreviations

AOR: Adjusted odds ratio
CI: Confidence interval
COR: Crude odds ratio
LLR: Likelihood ratio
RR: Relative risk
SNNPR: Southern Nations, Nationalities, and People's Region.
